# Enhanced maternal and child health nurse care for women experiencing intimate partner/family violence: protocol for MOVE, a cluster randomised trial of screening and referral in primary health care

**DOI:** 10.1186/1471-2458-12-811

**Published:** 2012-09-20

**Authors:** Angela J Taft, Rhonda Small, Cathy Humphreys, Kelsey Hegarty, Ruby Walter, Catina Adams, Paul Agius

**Affiliations:** 1 Associate Professor, Mother and Child Health Research, La Trobe University, Melbourne, Australia; 2 Professor/Director, Mother and Child Health Research, La Trobe University, Melbourne, Australia; 3 Professor, School of Social Work, University of Melbourne, Melbourne, Australia; 4 Associate Professor, Primary Care Research Unit, Department of General Practice, University of Melbourne, Melbourne, Australia; 5 School of Nursing, Victoria University, Melbourne, Australia; 6 Mother and Child Health Research, La Trobe University, Melbourne, Australia; 7 Statistician, Mother and Child Health Research, La Trobe University, Melbourne, Australia

**Keywords:** Intimate partner violence, Screening, Cluster randomised controlled trial, Maternal and child health nurse

## Abstract

**Background:**

Intimate partner violence (IPV) can result in significant harm to women and families and is especially prevalent when women are pregnant or recent mothers. Maternal and child health nurses (MCHN) in Victoria, Australia are community-based nurse/midwives who see over 95% of all mothers with newborns. MCHN are in an ideal position to identify and support women experiencing IPV, or refer them to specialist family violence services. Evidence for IPV screening in primary health care is inconclusive to date. The Victorian government recently required nurses to screen all mothers when babies are four weeks old, offering an opportunity to examine the effectiveness of MCHN IPV screening practices. This protocol describes the development and design of MOVE, a study to examine IPV screening effectiveness and the sustainability of screening practice.

**Methods/design:**

MOVE is a cluster randomised trial of a good practice model of MCHN IPV screening involving eight maternal and child health nurse teams in Melbourne, Victoria. Normalisation Process Theory (NPT) was incorporated into the design, implementation and evaluation of the MOVE trial to enhance and evaluate sustainability. Using NPT, the development stage combined participatory action research with intervention nurse teams and a systematic review of nurse IPV studies to develop an intervention model incorporating consensus guidelines, clinical pathway and strategies for individual nurses, their teams and family violence services. Following twelve months’ implementation, primary outcomes assessed include IPV inquiry, IPV disclosure by women and referral using data from MCHN routine data collection and a survey to all women giving birth in the previous eight months. IPV will be measured using the Composite Abuse Scale. Process and impact evaluation data (online surveys and key stakeholders interviews) will highlight NPT concepts to enhance sustainability of IPV identification and referral. Data will be collected again in two years.

**Discussion:**

MOVE will be the first randomised trial to determine IPV screening effectiveness in a community based nurse setting and the first to examine sustainability of an IPV screening intervention. It will further inform the debate about the effectiveness of IPV screening and describe IPV prevalence in a community based post-partum and early infant population.

**Trial registration:**

ACTRN12609000424202

## Background

Intimate partner violence (IPV) has been defined as any behaviour by an intimate partner which causes physical, sexual or psychological harm and IPV results in lasting health damage particularly for women and children [1]. Pregnant women and those with infants are especially vulnerable to IPV [2,3]. During pregnancy, IPV has been estimated at between four to eight percent. It can commence, continue or escalate in pregnancy and continue into the postpartum period [4] with serious physical and emotional consequences [5,6].

This study is located in Melbourne Australia, where IPV among women in the childbearing years is estimated to be the major contributor to the burden of illness (especially to mental health issues) amongst women of childbearing age. It is also a significant cause of adverse pregnancy outcomes [7-9]. IPV has a significant impact on women's parenting abilities, ultimately compromising their children's development [9]. Current research indicates that many children witnessing IPV can experience behavioural, emotional, developmental and psychobiological problems and difficulties with social competence in the longer term [10]. The damage can be incremental, but is also cumulative and while some children demonstrate resiliency, it is not yet clear why. Therapeutic services for mothers and their children who have experienced violence are now developing and offer valuable sources of referral if the problem is detected early and referrals made.

The potential for health services to support women and children experiencing IPV in the early infant years has been recognised with increasing government policies around screening for IPV in the perinatal periods [11]. However, the evidence of benefit for women and children of screening for IPV is scarce [12-14]. Nevertheless, in spite of this lack of evidence, screening all women is a common policy initiative and primary health care professionals are increasingly mandated to screen for IPV, especially in the childbearing years.

Health care professionals are commonly reluctant to ask about IPV and women may also be reluctant to disclose. Common barriers for health-care professionals’ inquiry have been found to be lack of professional training, support, resources and workload [15]. While there is evidence that training can increase clinician confidence, rates of identification and referral in a given study period, a major problem is the lack of evidence of the sustainability of health-care provider behaviour change and evidence for subsequent effective interventions [14]. This paper reports the protocol for an IPV screening randomised trial located in maternal and child health nurse clinics in Melbourne, Australia with an evaluation of the elements contributing to sustained screening practice.

### Screening for intimate partner violence (IPV)

IPV screening in health care systems can be defined as routine and systematic inquiry of all presenting women about their experience of intimate partner violence, using a consistent set of questions [16]. It remains a controversial subject as reviewers of the same evidence have come to different conclusions about its effectiveness, and as to whether it should be recommended [14,16-18]. Scholars have identified many barriers to universal IPV screening, including health care provider attitudes, heavy workloads, and lack of recurrent training, of adequate referral services and other resourcing support, and the presence of partners during consultations [14,19-22]. Nevertheless, governments are increasingly turning to universal screening as a policy option with varying levels of readiness to address the identified barriers to sustaining screening practices. There have been varying rates of screening coverage reported, depending on whether screening data are collected routinely or ‘snapshots’ are taken when service providers know their governments are scrutinizing performance at a particular time [16]. While routine screening is commonly assumed to be a desirable goal, a recent trial of an alternative approach, system supported ‘case finding’ or inquiry when IPV symptoms are present by primary care providers, was successful in raising identification and referral numbers, although benefits for women are unknown [23].

### The Victorian maternal and child health service and the context for IPCV screening

Victorian maternal and child health nurses (MCHNs) are trained nurse midwives who visit over 95% of all Victorian mothers with newborns at home and continue to offer support at their local centres until the children are six years old [24]. This free, universal, comprehensive primary health-care service aims to provide health promotion, illness prevention, early detection of developmental problems, and intervention to enhance the health and wellbeing of young children and their families. Australia has three tiers of government: federal or national; state (or territory) and local government or council. MCHN nursing is located and managed in the complex local government environment, while implementing policies formulated at state government level. They are often funded at local government level (usually 60% local government and 40% state government).

In late 2009, just as this proposed trial of IPV screening versus usual care was funded, a new comprehensive approach to screening children and their mothers for other common problems and developmental delays, Key Ages and Stages (KAS), was introduced into Victorian maternal and child health nursing practice [25]. Under the new Key Ages and Stages protocol, MCHNs are now required to screen all mothers for family violence - the government’s preferred term, which includes child abuse (not only IPV) - when their babies are four weeks old. MCHNs were provided with three hours training to use a Common Risk Assessment Framework, offering a common definition of IPV for health care providers, police, family violence services and courts, symptoms to look for and referral processes. Included in KAS strategies were computer prompts for the now mandated four week IPV screening, any screening at a later consultation, and the requirement to report regular data about IPV screening, safety plans and referrals and other child health screening and referral rates to government.

Nurses are required to ask the following questions about family violence:

1. Are you in any way worried about the safety of yourself or your children?

2. Are you afraid of someone in your family?

3. Has anyone in your household ever pushed, hit, kicked, punched or otherwise hurt you?

4. Would you like help with this now?

### MOSAIC (MOtherS' advocates in the community)

Prior to this government initiative, in our previous IPV trial of non-professional mother-to-mother support for pregnant or recent abused mothers, known as MOSAIC [26], MCH nurses struggled to identify and refer clients experiencing violence despite comprehensive six-hour training by a domestic violence training agency and additional resources (referral booklets, posters etc.). An impact evaluation conducted at the end of the study, showed that 66% of nurses said that they were comfortable or very comfortable asking about partner violence, while 18% remained uncomfortable. Thirty-five percent cited partners or family members being present as the major obstacle to asking about IPV, followed by fear of embarrassing women (14%) and the need to focus on the child as their priority (13%). When asked what made it difficult, 42% of nurses said women's reluctance to take further steps, referral agencies’ waiting lists (22%) and the difficulty of finding support for women with special needs (17%). When asked what should be put in place to assist other nurses to develop their skills and increase their confidence in this area, nurses suggested regular training (1); MCHN specific clinical guidelines for IPV (2); and an MCHN clinical pathway (3).

Following on from MCHN feedback in MOSAIC, this study originally aimed to compare implementation of IPV screening with usual care; however with the introduction of mandatory IPV screening of all recent mothers when babies are four weeks old, the trial design was modified to evaluate an enhanced model of IPV screening addressing some of the identified challenges for nurses and addressing the issue of sustainability, compared with basic mandatory IPV screening.

### Trial registration

This trial was registered with the Australian and New Zealand Clinical Trials Registry (ACTRN12609000424202).

### Ethics

This study was approved by the Human Ethics Committee, La Trobe University (UHEC 08–142) and also by the University of Melbourne and the Victorian Government Department of Education and Early Childhood Development.

### Aims

1) To compare whether, after implementing an evidence-based consensus model of good IPV practice for twelve months, MCH nurses in the intervention arm more frequently than nurses in the comparison arm

• Ask about IPV among their client population

• Refer women experiencing IPV to services

• Inquire about the safety of women and children in the relationship

2) More women attending MCH centres in the intervention arm than in the comparison arm:

• Are asked about IPV

• Disclose/discuss any abuse and

• Are satisfied with the quality of care and support they receive

3) To provide an accurate estimate of the proportion of MCH clients who have ever or who are currently experiencing IPV and those abused when pregnant

### The primary outcomes for the study are to

1. Increase the proportion of women (15%) reporting that they have been asked about family violence or nurses reporting they have screened for family violence (i.e. questions from the Key Ages and Stages protocol)

2. Increase the proportion of women disclosing IPV (discussing IPV with MCH nurse) or nurses reporting discussing safety plans

3. Increase the proportion of referrals reported by nurses or women reporting being offered referrals

### Secondary outcomes are to

1. Estimate the prevalence of any IPV in the previous 12 months (measured with the Composite Abuse Scale, ≥3-6, and ≥7), IPV during pregnancy and any reported child abuse

2. Increase the proportion of MCH clients reporting satisfaction with quality of care and support they receive for themselves, their partners and their children

3. Investigate the proportions of women reporting any harm arising from (a) responding to questions about IPV in their MCHN consultation or (b) responding to questions about IPV in the survey)

### Design

**Figure 1:**
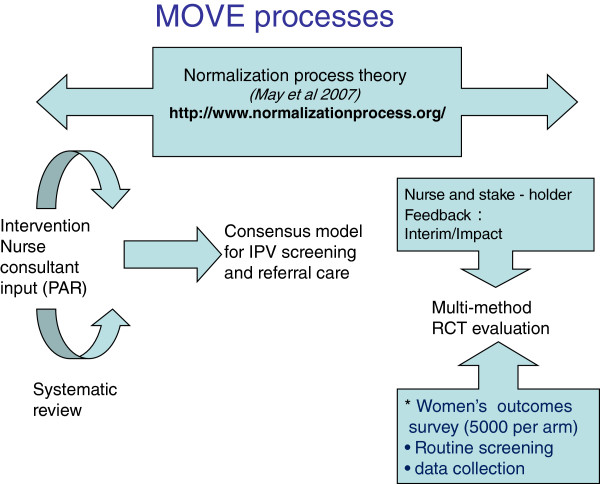
MOVE processes.

MOVE is a cluster randomised trial. It is located in MCHN teams in the north-west suburbs of Melbourne, consisting of 80 nursing centres and over 160 nurses (Figure 1).

#### Recruitment of clusters: MCH nurse teams in north west Melbourne

Team leaders of eight MCH teams, located in eight local government areas were invited to participate in a previous study MOSAIC [26] and all agreed. Each team leader and their manager signed a supplementary letter to the original Memorandum of Understanding to participate in this subsequent study.

#### Randomization

The randomization of nurse teams at that time occurred at a public randomization meeting. Names of the MCHN teams, stratified by numbers of births per local government area and paired in size, were concealed in opaque envelopes and the random selection made by an invited guest from outside the project or research team. MCH Team Leaders were present to ensure the fairness of the process and to check the contents of the envelopes prior to their being sealed. For MOVE, all eight teams agreed to the reverse of the previous randomisation allocation, so that teams allocated to comparison status in MOSAIC formed the intervention arm for MOVE and those previously allocated intervention status formed the comparison arm.

#### Blinding (allocation concealment)

Given the nature of the intervention, it has not been possible to blind MCHN teams or nurses to their status. The Project Coordinator is not blind to MCHN team participant status either, as she is required to liaise with intervention team MCHN consultants. Women attending the MCHN service are blind to their status as randomization is by cluster and consent to participate in the trial was given at MCH team level.

#### Blinding (outcome assessment)

All outcome and process evaluation data are self-completed by women and nurses and are anonymous:

(a) MCHN staff complete process and impact survey instruments on line using an anonymised format and data are stored in a secure database

(b) MCHN complete anonymised routine computerized screen data that are summarized annually and forwarded to the Department of Education and Early Childhood Development

(c) Names and addresses of women attending MCH clinics are not accessible to research staff because of privacy regulations. Surveys are sent to women in anonymised questionnaire booklets mailed to their home by a local government agency with an accompanying letter from their MCHN team leader. Outcomes were pre-specified at the time of our trial registration. Analyses are to be carried out by the study statistician blinded for trial arm following data entry of women’s survey responses by an independent data company.

#### Survey sample size calculation

(b) We calculated that around 8-10% of women attending each of the eight MCH services will have been abused in the previous 12 months [4]. Following twelve months implementation, we will send a mailed questionnaire to all women who have given birth in the previous 8 months in both the comparison and intervention municipalities (5000 per arm). This sample size enables us to detect a predicted increase of 15% average disclosure found previously in a screening intervention of screening vs usual care [27]. We have taken into account both the numbers of births in participating local government areas (using most recent published data (2005), the likely response fraction (55-65% following mailed surveys and a reminder postcard - a modified Dillman procedure) and also adjusted for clustering. We adjusted for an intra-cluster correlation of 0.02 from a previous IPV primary care study [28].

### Outcome measures

#### Routine data

Data reported routinely to the Victorian government is entered at the time of consultation by the MCH nurse. In relation to IPV screening, these data include:

• Whether the nurse reports asking about any of three family violence questions to which the answer is yes/no and at what time point: home visit; four weeks, eight weeks, four, eight, twelve or eighteen months.

• Whether the nurse reports making a safety plan

• Whether the nurse reports making a referral

We will compare rates of inquiry, safety plans and referrals between intervention and comparison arms at four weeks, four months and twelve months.

#### Survey measures

The survey booklet ‘Women’s Experiences of Health Care and Support with a New Baby’ has seven sections: (A) About your baby; (B) About your own health following your baby; (C) Your health care support; (D) Other care and support you receive; (E) Relationships and family; (F) You and your partner; (G) About you. It is designed to maximize women’s safety as it is mailed to her home. Section F includes questions about intimate partner violence measured using Hegarty’s Composite Abuse Scale [29], together with questions about being asked, disclosure (and comfort to disclose), rates of referral and satisfaction measures for herself, her children and in relation to strategies for her partner.

The questionnaire also includes depression and anxiety measures using the DASS-21 [30,31]; women’s experiences of motherhood measured with the Experience of Motherhood Scale [32] and a range of other assessments of women’s experiences.

#### Checklists

These data will only be available in the intervention arm, as they are a screening intervention tool (see below). Nurses use these screening self-completion forms with women and retain these for collection by MOVE research staff. They also record the screening consultation time (three or four months or later) and these will be analysed especially for the three month screening point, when routine data are not recorded.

## Methods

### Theoretical framework

An overarching goal of this study is the development of a sustainable model of MCHN good practice in identification, support and referral of women experiencing IPV. There has been considerable debate about the limitations of programs aimed at enhancing health care professional behavior change [33]. Further, it is now recognized that when interventions themselves are complex (such as the introduction of primary care nurse IPV screening) and operate in a complex environment (such as both state and local government arenas) that attention to theory, process and in particular, context are required if they are to be successfully integrated into routine practice [34]. This requires greater attention to the development, testing and refinement of an intervention before effectiveness testing.

May et al’s Normalization Process Theory [35-37] proposes four domains of work which require particular attention in the design, development, implementation and evaluation cycle of health system and health professional behavior change. We outline in brief how, in relation to the work of MCHN, we interpreted the core NPT concepts in relation to MCH nurse work and incorporated this into all aspects of the design, development, implementation, and process and impact evaluation of this trial. In terms of the core concepts, we interpreted them as related to MCH nursing work as follows:

• Coherence (how MCH nurses interpret, accept and value their role in, and the concept of IPV screening)

• Cognitive participation (how MCH nurses engage with the work: who introduces, leads, maintains and participates in IPV screening)

• Collective Action – involves four components:

∘ Interactional workability – how and when IPV screening is conducted, how screening roles are organized within teams, resources improving nurse/client screening interaction, who is allocated to the work

∘ Relational integration (accountability, knowledge and trust in IPV screening working relationships including team functioning and including nurse safety)

∘ Skill set workability (do MCH nurses think they have the required skills, resources and rewards to conduct IPV screening effectively)

∘ Contextual integration (how well is screening organized, funded and supported in their working context of local and state government)

• Reflexive monitoring ( how do MCH nurses and teams know how effective or well their IPV screening is going)

#### Intervention development phase – toward a model of sustainable good practice

This phase involved three strategies:

1.We conducted Participatory Action Research (PAR) with four volunteer MCHN consultants (one from each of four intervention MCHN teams) who explored the barriers and sought answers to these problems within their teams. PAR has been defined as ‘collective, self reflective inquiry that researchers and participants undertake, so they can understand and improve upon the practices in which they participate and the situations in which they find themselves’ [38]. Over a six-month period, at regular monthly meetings with MOVE staff, questions derived from earlier feedback in MOSAIC [26], discussion about barriers and enablers of IPV screening practices, and informed by the NPT framework [36] were agreed and nurse consultants took them away to their teams for discussion and feedback. The two groups (nurse consultants and MOVE staff) met over a six month period to discuss team feedback and reach agreement on the major aspects of a good practice model to be outlined in clinical pathways and guidelines. All meetings were recorded on tape and notes derived from them informed further discussion. We also drew on a GP systems intervention then being piloted in the UK (IRIS [23]) for strategies to strengthen links between clinicians and family violence services.

2. A systematic review of interventions (with controlled or comparison designs), guidelines or protocols targeted at improving the response of health care practitioners (HCPs) working with women experiencing IPV, their children and partners using abusive behaviors was conducted using Medline, Cochrane and Campbell Collaboration databases and CINAHL. Further details of the search terms and inclusion/exclusion criteria are available from the authors. Results were synthesized and presented to the nurse consultant and research group and the advisory group of key stakeholders.

3. Using a method we have previously used in an international collaboration to develop GP guidelines [39], we facilitated multidisciplinary group discussions between the nurse consultants and study reference group to independently score and then reach agreement about the content and format of the guidelines, clinical pathway and maternal health checklist – a woman’s self-completion screening tool (all available at http://www.latrobe.edu.au/mchr/html/move.html ). A penultimate draft of these materials was presented and finalized at a reference group meeting and further detail of the model’s key elements are presented below.

### Screening time

#### The move intervention model

MCH nurses expressed concern about an early screening time (e.g. home visit or four weeks) for the following reasons: (a) women were still recovering from the birth and focused their attention on the baby’s not their own needs; (b) nurses had had insufficient time to establish a trusting relationship with the woman which would make it easier to ask a sensitive question and enable disclosure (c) >30% of Victorian women have had Caesarean section and advised not to drive for six weeks, so are frequently accompanied by someone, making it sometimes challenging to ensure screening safely. Nurses therefore suggested that the optimum screening time would be at a three or four month visit when women could be advised ahead of time that the visit would be focused on their needs and that they would be completing a women’s health checklist. They also suggested that they could ask women not to bring other children if possible; and told accompanying partners that the questions would include discussion of menstruation and breast problems, and that partners may not want to be present. Two teams created an additional woman-focused three month visit, and the other two teams added fifteen minutes to the four month KAS-funded visit to be focused on maternal needs.

### Maternal health checklist

Evidence from the systematic review suggested that women preferred self-completion screening methods (e.g. paper or computer based) rather than face-to-face screening inquiry (ref). The maternal health checklist was developed as a tear-off pad consisting of an A-5 set of questions for women to self-complete at the agreed consultation which focused on maternal health, rather than the development of the baby, who is the MCH nurses’ primary client. When women arrived at this visit, they were given the checklist and asked to complete it in a quiet corner and let the nurse know when finished. The checklist consists of a set of questions about maternal health, beginning with questions about physical health, such as headache, nipple or bowel problems then moving to those about depression, contraception or drug misuse to the same family violence questions being asked as part of the Key Ages and Stages (KAS) mandatory screening at four weeks. After discussion, MOVE added two further questions to this list. Just before the KAS questions, we added:

*Do you have any problems in your relationships or intimacy with your partner?* and *Has anyone in your household ever humiliated you or tried to control what you can or cannot do?* The first question was seen as useful for women who may want to discuss non-specific problems in the relationship prior to disclosure and the latter because it is central to intimate partner abusive behavior. If a woman ticked any of the relationship or any family violence questions, nurses were asked to initiate a discussion about these in ways that felt appropriate and comfortable. The completed checklist form is retained as part of the study data collection.

### Clinical guidelines and pathway

The eight-page guidelines were designed so that the clinical pathway was on the front page and summarized recommended actions and also feature principles of good practice along the bottom.

These principles are:

• SEEK SUPPORT Consult your team leader, nurse mentor, family violence liaison worker.

• MAINTAIN PRIVACY & CONFIDENTIALITY

• SAFETY FIRST of nurse, women & children

• BE NON JUDGEMENTAL

• MAINTAIN PROFESSIONAL BOUNDARIES your role is to listen, support & refer

• MINDFULNESS Stages of change. Where is the woman up to?

Further inside are four sections entitled:

• **Inquire and Connect** (this section deals with processes for screening including cross-cultural practice and nurse safety – a nursing concern)

• **Assess and Support** (this section deals with the processes following disclosure and includes a section aiming to alert the nurse to the fact that not all women are ready either to disclose or to accept referrals. The section stresses asking about women’s circumstances, supportive listening and was developed with the knowledge that some nurses had difficulty with accepting women’s decision not to leave when they were unready. It also outlines ‘warm referrals’ and gives advice about clinical decision-making when children are at risk

• **Document** (together with the Women’s Legal Resource Service, MOVE developed a guide to objective documentation which would be useful for court purposes should a nurse be required to give evidence in court)

• **Quality Assurance and Routine Practice** (this section includes routine team monitoring, support for nurses experiencing family violence and mindful practice.

Each section outlines the Aims, Guidelines and Implementation issues, and details practice, including which type of nurse – the universal nurse, the nurse mentor or coordinator/team leader – has responsibility for ensuring implementation.

### Nurse mentors

Nurse mentors volunteer or were chosen by team leaders as those nurses with a special interest in and/or skills in family violence practice. They provide support to individual nurses in the team struggling with difficult cases or wanting to practice their IPV inquiry skills. They act as a ‘practice champion’ in the team.

The role of these nurses includes:

• Liaising with the family violence liaison officers in community-based family violence services

• Accompanying MCHNs who are undertaking a home visit for additional safety where there is or is likely to be IPV

• Facilitating and maintaining discussions within the team about IPV issues

• Acting as a secondary referral, debriefing and support person for nurses managing difficult cases of IPV

### Family violence MCHN liaison workers

MOVE adopted the model outlined in the IRIS study [20] of a family violence agency staff member who has a special responsibility for liaison with primary care services; in this case, with the MCH nurse teams to strengthen links and referral options. These staff are funded, employed and managed by the family violence services, with agreed responsibility during the trial for:

• Attending MCHN team meetings to familiarize the team with the FV service and other relevant community-based services, e.g. community police

• Facilitating referrals and secondary referrals to family violence services

• Providing feedback about referrals, debriefing if necessary

• Participating in evaluation interviews

The geographical location of the two family violence services resulted in one family violence liaison worker liaising with one team and the other liaising with three teams.

### Implementation

Following all stakeholders’ agreement with the new model, nursing consultants and MOVE staff briefed the intervention teams and provided them all with newly developed resources (http://www.latrobe.edu.au/mchr/html/move.html) including a guide to IPV documentation. Teams undertook to implement the MOVE model over a twelve month period. During the intervention period, MOVE research staff remained at a distance to enhance sustainability, and reliance on research staff was kept to a minimum.

### Data collection

*Process evaluation*: Online survey questions for nurses, structured using tenets of the Normalization Process Theory [36] were developed in consultation with nurse consultants, face validity was checked with assessment by stakeholders in the reference group and they were then piloted with an MCH nurse team outside the trial to ensure acceptability, relevance and timeliness.

The online survey was developed for use six months into the implementation year with all participating nurses in both arms to elicit MCHN current IPV screening knowledge, attitudes and work practices.

At the same time, telephone or face-to-face interviews were planned with MCH nurse mentors, team leaders, family violence liaison officers and supervising staff from family violence agencies to elicit factors related to key concepts of the NPT framework. Interviews were conducted by external staff.

#### *Impact evaluation* - o*nline survey data*

Three months after the end of the intervention, a second anonymous online survey of MCH nurses in both arms of the study (~160) was developed to assess their levels of confidence about and attitudes to IPV screening, perceived rates of inquiry about women’s and children’s safety, referral to family violence agencies for all family members, team functioning and support for IPV screening and perceptions of their own safety. The surveys and interviews will be repeated two years after the end of the intervention to assess sustainability of the screening processes. Interviews conducted by external staff with key stakeholders will also be conducted at the end of the intervention and two years later.

#### Outcome evaluation - primary and secondary outcomes

##### Routine data

With local government permission, we will retrieve routine data about family violence described above for the intervention period for all nurses in both arms of the study. We will seek to retrieve it again in 2013, two years post intervention.

##### Women’s survey questionnaire

We have developed a questionnaire survey to be sent to 10,000 women (5000 in either arm) who have given birth over an eight month period. This sample gives us power to detect differences in the primary outcome. Included are all the primary and secondary outcome measures and a valid and reliable measure of intimate partner violence (Composite Abuse Scale) [29].

The data management company employed to manage the survey data has been subcontracted to the local government councils who have given the company staff access to women client’s addresses. They will mail the surveys and two rounds of reminder cards. Surveys will be returned to the company for electronic data entry and double-coding.

##### Checklists (http://www.latrobe.edu.au/mchr/html/move.html)

MOVE staff will retrieve all checklists retained by intervention nurses at intervention midpoint (six months) and after the implementation period has finished. Data will be checked for completion of screen and screening period.

### Data analysis and reporting

#### Women’s survey

The MCH consumer survey data will be cleaned, double-coded and entered into a secure database by an external company blinded to the study arms. It will then be forwarded to a statistician blinded to study arm. Women’s characteristics for socio-demographic and birth characteristics will be compared by trial arm to ensure that randomization was effective. The representativeness of the women responding to the survey (i.e. sample bias due to differential study participation rates) will be assessed by comparison with routine perinatal data for all women giving birth in the region for the comparison year (Victorian Perinatal Data Collection Unit, DHS).

All data will be analyzed by intention-to-treat and variance estimates in models will be adjusted for the effect of the clustered (LGA) selection of participants. We will analyze data using contingency table analyses for bivariate and binary logistic regression with robust standard errors for multivariate models. For primary outcomes, we will analyze data stratified by and adjusted for women’s abuse status and for potentially significant confounding variables (where randomization is less effective in removing bias across study groups), including women’s socio-economic status.

##### For MOVE primary outcomes

we will report:

• Any difference in proportions by trial arm (from survey and routine data) of screening for IPV (questions about fear of a partner; physical abuse and concern about the safety of their children) will be reported separately and as a screening inquiry composite variable

• Any difference in proportions by trial arm of women reporting IPV (Composite Abuse Scale ≥ 3 and ≥7) who have discussed IPV with their MCH nurse

• Any difference in proportions by trial arm of nurses who have reported that they have made safety plans with women clients

• Any difference in proportions by trial arm of women reporting IPV (Composite Abuse Scale ≥ 3 and ≥7) who have been offered a referral (if sufficient numbers) or nurses who report they have made referrals for IPV (routine data)

The following measures will be reported for the overall population and also by trial arm.

• Any difference in proportions by trial arm of abused and non-abused women reporting satisfaction with their MCH nursing care

• Intimate partner violence measured by the Composite Abuse Scale reported for women with scores ≥ 3 and those with scores ≥ 7

• Proportions ever having been afraid and currently afraid of their partner

• Proportions reporting partner abuse during pregnancy for the recent pregnancy and any in a past pregnancy.

• Proportions abused in pregnancy by family members and by other known persons (e.g. employer, housemate)

• Proportions physically, sexually, emotionally abused or neglected as children

• Proportions reporting harm from IPV screening or participating in an IPV screening study

#### Process and impact evaluation

Online survey data will be downloaded by MOVE staff from the online survey database and entered into an Excel spreadsheet. Responses will be grouped by MCHN nurse team and separated into trial arms. Responses will be counted and proportions of responses for each questions calculated. These data will be compared across trial arm and analyzed using bivariate chi-square tests of independence or other non-parametric analytical approaches where appropriate.

Qualitative data (interviews with key stakeholders) will be transcribed verbatim, coded thematically exploring NPT constructs and for any other significant patterns and any outliers.

#### Dissemination and translation

We propose to present the findings from this study to Victorian and Australian policymakers at relevant national conferences, in person to the Victorian Government Department responsible for MCH nursing, at international IPV and nursing conferences, in peer-reviewed and professional journals.

### Two year follow-up

We propose to seek permission to obtain routine data for screening inquiry, safety plans and referrals for IPV two years after the MOVE implementation completion. We will also aim to conduct a survey with all nurses in the two arms of the MOVE trial to investigate the sustainability of the screening intervention, using NPT constructs. Analyses of these data will be conducted as outlined above.

## Discussion

MOVE will be the first randomised trial to determine IPV screening effectiveness in a community based nurse setting and the first to examine sustainability of an IPV screening intervention. Screening for IPV remains a controversial area, but governments especially in high and middle income countries continue to implement screening policy in health care settings, without addressing the key barriers reported by primary care professionals, especially workloads, ongoing training and lack of effective multi-agency collaboration. Without addressing such barriers, there is doubt about the sustainability or effectiveness of screening [27]. Normalisation Process Theory [36] has been developed to support sustainability of implementation and evaluation of complex interventions and complex settings and is appropriate to the effectiveness and sustainability questions addressed by the MOVE trial.

This study is being conducted in Victoria, Australia where legal sanctions and strong family violence policies exist at national and state level. As a high income country crisis and outreach responses for women are funded and supported (although stretched to meet need), improved policing responses and intervention/protection orders available from magistrates courts tailored to family violence victims are also available. There are also behaviour change groups for male perpetrators and services for children who have experienced family violence. In this context, when primary care providers identify women experiencing family violence, services exist to support women and sanctions are available (however limited) to prevent further harm. As a result of previous training conducted in the MOSAIC study and the further training offered in the new Victorian Key Ages and Stages initiative, MOVE nurse teams in both arms of this study have been well trained to screen and refer women experiencing violence. Of all Victorian MCH nurses, nurses in this study are well prepared and the intervention arm nurses even better prepared, following their active involvement in developing a good practice model.

MOVE results will further inform the debate about the effectiveness of IPV screening and describe IPV prevalence in a community based post-partum and early infant population.

## Competing interests

The author(s) declare that they have no competing interests.

## Authors’ contributions

AT designed the study, secured the funding and is responsible for overall MOVE coordination. She drafted and edited this paper. RS, CH and KH contributed to the design of the study and edited this paper. RW undertook the systematic review and CA designed the guidelines, pathway and maternal checklist. Both CA and RW contributed to the design and coordination of the intervention and edited this paper. PA designed the statistical analysis in consultation with AT and RS and edited this paper. All authors read and approved the final manuscript.

## Pre-publication history

The pre-publication history for this paper can be accessed here:

http://www.biomedcentral.com/1471-2458/12/811/prepub

## References

[B1] Krug EG World report on violence and health 2002 Geneva: WHO 346

[B2] Gazmararian J Prevalence of violence against pregnant women JAMA 1996 275 24 1915 1920 10.1001/jama.1996.03530480057041 8648873

[B3] Saltzman LE Physical Abuse around the Time of Pregnancy: an Examination of Prevalence and Risk Factors in 16 States Matern Child Health J 2003 7 1 31 43 10.1023/A:1022589501039 12710798

[B4] Martin SL Physical abuse of women before, during and after pregnancy JAMA 2001 285 12 1581 1584 10.1001/jama.285.12.1581 11268265

[B5] Brown SJ Fecal incontinence during the first 12 months postpartum: complex causal pathways and implications for clinical practice Obstet Gynecol 2012 119 2, Part 1 240 249 10.1097/AOG.0b013e318242b1f7 22270274

[B6] Campbell JC Health consequences of intimate partner violence Lancet 2002 359 1331 1336 10.1016/S0140-6736(02)08336-8 11965295

[B7] Taft A Watson L Lee C Violence against young Australian women and associated reproductive events: a cross sectional analysis Aust NZ J Public Health 2004 28 4 324 329 10.1111/j.1467-842X.2004.tb00438.x 15704695

[B8] Vos T Measuring the impact of intimate partner violence in the health of women in Victoria, Australia Bulletin of the World Health Organisation 2006 84 9 739 744 10.2471/BLT.06.030411 PMC2627471 17128344

[B9] McCosker-Howard H Woods AB Roberts G, Hegarty K, Feder G How is intimate partner abuse experienced by childbearing women, in Intimate partner abuse and health professionals: new approaches to domestic violence 2006 London: Churchill Livingstone, Elsevier 111 126

[B10] Smith JL Roberts G, Hegarty K, Feder G The impact of intimate partner violence on children Intimate partner violence and health professionals 2005 London, UK: Elsevier 127 144

[B11] Family Violence Prevention Fund National Consensus Guidelines on Identifying and Responding to Domestic Violence Victimisation in Health Care Settings 2004 San Francisco: Family Violence Prevention Fund 70

[B12] Moracco KE Cole TB Preventing intimate partner violence: screening is not enough JAMA 2009 302 5 568 569 10.1001/jama.2009.1135 19654392

[B13] Hooker L Ward B Verrinder G Domestic violence screening in maternal and child health nursing practice: a scoping review Contemp Nurse 2012 42 2 10.5172/conu.2012.42.2.198 23181372

[B14] Feder G How far does screening women for domestic (partner) violence in different health-care settings meet criteria for a screening programme? Systematic reviews of nine UK National Screening Committee criteria Health Technol Assess 2009 13 16 1 113 10.3310/hta13160 19272272

[B15] Hegarty K Feder G Ramsey J Roberts G, Hegarty K, Feder G Identification of intimate partner abuse in health care settings: should health professionals be screening? Intimate Partner Abuse and Health Care Professionals: New approaches to domestic violence 2006 London, UK: Churchill Livingstone (Elsevier) 79 92

[B16] Spangaro J Poulos RG Zwi AB Pandora Doesn’t Live Here Anymore: Normalization of Screening for Intimate Partner Violence in Australian Antenatal, Mental Health, and Substance Abuse Services Violence Vict 2011 26 1 130 144 10.1891/0886-6708.26.1.130 21776834

[B17] Nelson HD Bougatsos C Blazina I Screening Women for Intimate Partner Violence. A Systematic Review to Update the 2004 U.S. Preventive Services Task Force Recommendation Ann Intern Med 2012 156 11 10.7326/0003-4819-156-11-201206050-00447 22565034

[B18] Taket A Wathen N M H Should Health Professionals Screen All Women for Domestic Violence PLoS Med 2004 1 1 7 10 10.1371/journal.pmed.0010007 PMC523827 15526052

[B19] Chamberlain L Perham-Hester KA The impact of perceived barriers on primary care physicians' screening practices for female partner abuse Women Health 2002 35 2–3 55 69 12201510 10.1300/J013v35n02_04

[B20] Gregory A Primary care identification and referral to improve safety of women experiencing domestic violence (IRIS): protocol for a pragmatic cluster randomised controlled trial BMC Publ Health 2010 10 54 10.1186/1471-2458-10-54 PMC2825222 20122266

[B21] Hegarty KL Taft AJ Overcoming the barriers to disclosure and inquiry of partner abuse for women attending general practice Aust NZ J Public Health 2001 25 5 433 437 11688623

[B22] Waalen J Screening for Intimate Partner Violence by Health Care Providers Am J Prev Med 2000 19 4 230 237 10.1016/S0749-3797(00)00229-4 11064226

[B23] Feder G Identification and Referral to Improve Safety (IRIS) of women experiencing domestic violence with a primary care training and support programme: a cluster randomised controlled trial Lancet 2011 378 1788 1795 10.1016/S0140-6736(11)61179-3 22000683

[B24] Department of Education and Early Childhood Development (DEECD) Maternal and child health service: Practice Guidelines 2009 Melbourne, Victoria: State Government of Victoria

[B25] Department of Education and Early Childhood Development (DEECD) Maternal and Child Health Service: Key Ages and Stages Framework 2009 Melbourne: State Government of Victoria

[B26] Taft AJ MOSAIC (MOtherS' Advocates In the Community): protocol and sample description of a cluster randomised trial of mentor mother support to reduce initmate partner violence among pregnant or recent mothers BMC Publ Health 2009 9 159 10.1186/1471-2458-9-159 PMC2702379 19473534

[B27] Stayton CD MM D Mutable influences on Intimate Partner Abuse Screening in Health Care Settings: a Synthesis of the Literature. Trauma Violence and Abuse 2005 6 271 271 285 10.1177/1524838005277439 16217117

[B28] Hegarty K Physical and social predictors of partner abuse in women attending general practice Br J Gen Pract 2008 58 484 487 10.3399/bjgp08X299245 18611314 PMC2441509

[B29] Hegarty K Bush R Sheehan M The composite abuse scale: further development and assessment of reliability and validity of a multidimensional partner abuse measure in clinical settings Violence Vict 2005 20 5 529 547 16248489

[B30] Lovibond SH Lovibond PF Manual for the Depression Anxiety Stress Scales 1995 2 Sydney: Psychology Foundation

[B31] Osman A The Depression Anxiety Stress Scales-21 (DASS-21): Further Examination of Dimensions, Scale Reliability, and Correlates J Clin Psychol 2012 10.1002/jclp.21908. [Epub ahead of print] 22930477

[B32] Astbury J Making motherhood visible: The experience of motherhood questionnaire Journal of Reproductive and Infant Psychology 1994 12 2 79 88 10.1080/02646839408408871

[B33] Colombini M Mayhew S Watts C Health-sector responses to intimate partner violence in low- and middle-income settings: a review of current models, challenges and opportunities Bull World Health Organ 2008 86 8 635 642 10.2471/BLT.07.045906 18797623 PMC2649453

[B34] Gunn JM Embedding effective depression care: using theory for primary care organisational and systems change Implement Sci 2010 5 10.1186/1748-5908-5-62 PMC2925331 20687962

[B35] May C A rational model for assessing and evaluating complex interventions in health care BMC Health Services Research 2006 6 1 86 10.1186/1472-6963-6-86 16827928 PMC1534030

[B36] May CR Process evaluation for complex interventions in primary care: understanding trials using the normalization process model. BMC BMC Family Practice 2007 8 1 42 10.1186/1471-2296-8-42 17650326 PMC1950872

[B37] May CR Evaluating complex interventions and health technologies using normalization process theory: development of a simplified approach and webenabled toolkit BMC Health Serv Res 2011 11 10.1186/1472-6963-11-245 PMC3205031 21961827

[B38] Baum F The New Public Health: An Australian Perspective 1998 Melbourne: OUP 569

[B39] Taft A Hegarty K Feder G Management of the whole family when intimate partner violence is present: guidelines for family clinicians 2006 RACGP: Melbourne

